# Supplementary material for: Effects of digital cognitive behavioral therapy for depression on suicidal thoughts and behavior: Protocol for a systematic review and meta-analysis of individual participant data
Source: PLoS One. 2023 Jun 8;18(6):e0285622. doi: 10.1371/journal.pone.0285622 (PMC10249902; doi:10.1371/journal.pone.0285622)
Supplement: Attachment — Submitted filename: PONE-D-23-05433_reviewer copy.pdf [file pone.0285622.s002.pdf]

PLOS ONE

Effects of digital depression interventions on suicidal thoughts and behavior: Protocol for a systematic review and meta-analysis of individual participant data

--Manuscript Draft--

**Manuscript Number:**	PONE-D-23-05433
**Article Type:**	Study Protocol
**Full Title:**	Effects of digital depression interventions on suicidal thoughts and behavior: Protocol for a systematic review and meta-analysis of individual participant data
**Short Title:**	Effects of digital depression interventions on suicidal thoughts and behavior
**Corresponding Author:**	Lasse Bosse Sander<br>Albert-Ludwigs-Universität Freiburg: Albert-Ludwigs-Universitat Freiburg<br>Freiburg, GERMANY
**Keywords:**	depression; Suicid*; Digital Interventions; Individual Patient Meta-Analysis
**Abstract:**	Introduction<br>Digital interventions for the treatment of depression have been extensively studied and shown to be effective in the reduction of depressive symptoms. However, little is known about their effects on suicidal thoughts and behaviors (STB). Information on the impact of digital interventions on STB are essential for patients' safety because most digital interventions are built as self-help interventions without direct support options in case of a suicidal crisis. We, therefore, aim to conduct a meta-analysis of individual participant data (IPD) to investigate the effects of digital depression interventions on STB and to explore potential effect moderators.<br>Methods<br>Data will be retrieved from an established and annually updated IPD database of randomized controlled trials investigating the effectiveness of digital cognitive behavior therapy interventions for depression in adults and adolescents. We will conduct a one-stage and a two-stage IPDMA on the effects of these interventions on STB. All types of control conditions are eligible. STB can be measured with specific scales (e.g., Beck scale suicide, BSS) or single items from depression scales (e.g., item 9 of the PHQ-9) or standardized clinical interviews. Multilevel linear regression will be used for specific scales, and multilevel logistic regression will be used for treatment response or deterioration, which will be operationalized as a change in score by at least one quartile from baseline. Moderator analyses will be conducted at the participant-, study-, and intervention-level. Two independent reviewers will assess the risk of bias using the Cochrane Risk of Bias Tool 2.<br>Conclusion<br>This IPD meta-analysis will harness the available data to assess the effects (response and deterioration) of digital depression interventions on STB. Information about changes in STB is essential to estimate patients'safety when engaging in digital treatment formats.
**Order of Authors:**	Lasse Bosse Sander
	Marie Beisemann
	Eirini Karyotaki
	Wouter van Ballegooijen
	Pim Cuijpers
	Tobias Teismann
	Philipp Doebler
	Matthias Domhardt
	Harald Baumeister
	Rebekka Büscher
**Additional Information:**	

Powered by Editorial Manager® and ProduXion Manager® from Aries Systems Corporation

Question	Response
**Financial Disclosure**<br><br>Enter a financial disclosure statement that describes the sources of funding for the work included in this submission. Review the submission guidelines for detailed requirements. View published research articles from *PLOS ONE* for specific examples.<br><br>This statement is required for submission and **will appear in the published article** if the submission is accepted. Please make sure it is accurate.<br><br>**Unfunded studies**<br>Enter: *The author(s) received no specific funding for this work.*<br><br>**Funded studies**<br>Enter a statement with the following details:<br>• Initials of the authors who received each award<br>• Grant numbers awarded to each author<br>• The full name of each funder<br>• URL of each funder website<br>• Have or will the sponsors or funders play any role in the study design, data collection (when applicable) and analysis, decision to publish, or preparation of the manuscript?<br>• **NO** - Include this sentence at the end of your statement: *The funders did not and will not have a role in study design, data collection and analysis, decision to publish, or preparation of the manuscript.*<br>• **YES** - Specify the role(s) played.<br><br>* typeset	LBS received funding for the study described in this protocol from the German Research Foundation (project “Effects of internet interventions for depression on suicide ideation - a meta-analysis of individual participant data (IPDMA), grant No. SA 3767/2-1).<br>http://www.dfg.de<br>The funders had no role in the study design, the decision to publish, or in the preparation of the manuscript. The views expressed are those of the author(s) and not necessarily those of the funder. The article processing charge was funded by the German Research Foundation (DFG) and the University of Freiburg in the funding program Open Access Publishing.
**Competing Interests**<br><br>Use the instructions below to enter a competing interest statement for this submission. On behalf of all authors, disclose any competing interests that could be perceived to bias this work—acknowledging all financial support and any other relevant financial or non-financial competing interests.	The authors have declared that no competing interests exist.

This statement is required for submission and **will appear in the published article** if the submission is accepted. Please make sure it is accurate and that any funding sources listed in your Funding Information later in the submission form are also declared in your Financial Disclosure statement.<br><br>View published research articles from *PLOS ONE* for specific examples.<br><br>**NO authors have competing interests**<br><br>Enter: *The authors have declared that no competing interests exist.*<br><br>**Authors with competing interests**<br><br>Enter competing interest details beginning with this statement:<br><br>*I have read the journal's policy and the authors of this manuscript have the following competing interests: [insert competing interests here]*<br><br>* typeset	
**Ethics Statement**<br><br>Enter an ethics statement for this submission. This statement is required if the study involved:<br><br>• Human participants<br>• Human specimens or tissue<br>• Vertebrate animals or cephalopods<br>• Vertebrate embryos or tissues<br>• Field research<br><br>Write "N/A" if the submission does not require an ethics statement.<br><br>General guidance is provided below. Consult the submission guidelines for detailed instructions. **Make sure that all information entered here is included in the Methods section of the manuscript.**	The ethics committee of the German Psychological Association (Deutsche Gesellschaft für Psychologie, DGPS) approved this study in a written consent.<br>Approval number: SanderLasse2020-04-08DM.

**Format for specific study types**<br><br>**Human Subject Research (involving human participants and/or tissue)**<br>• Give the name of the institutional review board or ethics committee that approved the study<br>• Include the approval number and/or a statement indicating approval of this research<br>• Indicate the form of consent obtained (written/oral) or the reason that consent was not obtained (e.g. the data were analyzed anonymously)<br><br>**Animal Research (involving vertebrate animals, embryos or tissues)**<br>• Provide the name of the Institutional Animal Care and Use Committee (IACUC) or other relevant ethics board that reviewed the study protocol, and indicate whether they approved this research or granted a formal waiver of ethical approval<br>• Include an approval number if one was obtained<br>• If the study involved *non-human primates*, add *additional details* about animal welfare and steps taken to ameliorate suffering<br>• If anesthesia, euthanasia, or any kind of animal sacrifice is part of the study, include briefly which substances and/or methods were applied<br><br>**Field Research**<br><br>Include the following details if this study involves the collection of plant, animal, or other materials from a natural setting:<br>• Field permit number<br>• Name of the institution or relevant body that granted permission	
**Data Availability**<br><br>Authors are required to make the data underlying their research findings fully available, without restriction.<br><br>If the manuscript reports pilot or preliminary results, the data underlying those results must be made available at	N/A - No results are reported

Powered by Editorial Manager® and ProduXion Manager® from Aries Systems Corporation

the time of publication. A Data Availability Statement describing where the data can be found is required at submission. Your answers to this question constitute the Data Availability Statement and will be published in the article, if accepted. When legal or ethical requirements prohibit public data sharing, authors must indicate how researchers may obtain access to the data. See the PLOS Data Policy and FAQ for detailed information.<br><br>**Important:** Stating 'data available on request from the author' is not sufficient. If your data are only available upon request, select 'No' for the first question and explain your situation in the text box.<br><br>For protocols without pilot or preliminary data, authors are strongly encouraged to state how they plan to share research data from their study when it is completed or published.<br><br>If your study includes pilot data, will they be made fully available without restriction?	
**Describe where the data may be found in full sentences. If you are copying our sample text, replace any instances of XXX with the appropriate details.**<br><br>• If **no pilot data are reported** in the manuscript, please enter the following: *No datasets were generated or analysed during the current study. All relevant data from this study will be made available upon study completion.*<br>• If the manuscript reports pilot data **held in a public repository**, include URLs, accession numbers or DOIs. If this information will only be available after acceptance, indicate this by ticking the box below. For example: *All XXX files are available from the XXX database (accession number(s) XXX, XXX.).*<br>• If the manuscript reports pilot data	The analysis R codes will be made<br>available online at OSF with publication. Individual participant<br>data cannot be provided due to confidenciality agreements.

contained **within the manuscript and/or Supporting Information files**, enter the following:<br>*All relevant data are within the manuscript and its Supporting Information files.*<br>• If neither of these applies but you are able to provide **details of access elsewhere**, with or without limitations, please do so. For example:<br><br>*Data cannot be shared publicly because of [XXX]. Data are available from the XXX Institutional Data Access / Ethics Committee (contact via XXX) for researchers who meet the criteria for access to confidential data.*<br><br>*The data underlying the results presented in the study are available from (include the name of the third party and contact information or URL).*<br>• This text is appropriate if the data are owned by a third party and authors do not have permission to share the data.<br><br>For study protocols with data management or sharing plans, authors are encouraged to briefly describe in their Data Availability Statement how data they generate will be made accessible when the study is completed. For example:<br>• Deidentified research data will be made publicly available when the study is completed and published.<br><br>* typeset	
Additional data availability information:	Tick here if the URLs/accession numbers/DOIs will be available only after acceptance of the manuscript for publication so that we can ensure their inclusion before publication.

Manuscript
Click here to access/download;Manuscript;Sander_et_al_IPDMA_protocol.d

Protocol

Effects of digital depression interventions on suicidal thoughts and behavior: Protocol for a systematic review and meta-analysis of individual participant data

Lasse B. Sander[1,2], Marie Beisemann[3], Eirini Karyotaki[4], Wouter van Ballegooijen[4,5], Pim Cuijpers[4], Tobias Teismann[6], Philipp Doebler[3], Matthias Domhardt[7], Harald Baumeister[7], Rebekka Büscher[1]

Affiliations

1 Medical Psychology and Medical Sociology, Faculty of Medicine, University of Freiburg, Freiburg, Germany

2 Black Dog Institute, University of New South Wales, Sydney, NSW, Australia

3 Department of Statistics, TU Dortmund University, Dortmund, Germany

4 Department of Clinical, Neuro and Developmental Psychology, Amsterdam Public Health Research Institute, Vrije Universiteit Amsterdam, Amsterdam, The Netherlands

5 Department of Psychiatry, Amsterdam UMC, Amsterdam, The Netherlands

6 Mental Health Research and Treatment Center, Faculty of Psychology, Ruhr-Universität Bochum, Germany

7 Institute of Psychology and Education, Department of Clinical Psychology and Psychotherapy, Ulm University, Ulm, Germany

Word count: 3356

Abstract

Introduction

Digital interventions for the treatment of depression have been extensively studied and shown to be effective in the reduction of depressive symptoms. However, little is known about their effects on suicidal thoughts and behaviors (STB). Information on the impact of digital interventions on STB are essential for patients' safety because most digital interventions are built as self-help interventions without direct support options in case of a suicidal crisis. We, therefore, aim to conduct a meta-analysis of individual participant data (IPD) to investigate the effects of digital depression interventions on STB and to explore potential effect moderators.

Methods

Data will be retrieved from an established and annually updated IPD database of randomized controlled trials investigating the effectiveness of digital cognitive behavior therapy interventions for depression in adults and adolescents. We will conduct a one-stage and a two-stage IPDMA on the effects of these interventions on STB. All types of control conditions are eligible. STB can be measured with specific scales (e.g., Beck scale suicide, BSS) or single items from depression scales (e.g., item 9 of the PHQ-9) or standardized clinical interviews. Multilevel linear regression will be used for specific scales, and multilevel logistic regression will be used for treatment response or deterioration, which will be operationalized as a change in score by at least one quartile from baseline. Moderator analyses will be conducted at the participant-, study-, and intervention-level. Two independent reviewers will assess the risk of bias using the Cochrane Risk of Bias Tool 2.

Conclusion

This IPD meta-analysis will harness the available data to assess the effects (response and deterioration) of digital depression interventions on STB. Information about changes in STB is essential to estimate patients' safety when engaging in digital treatment formats.

Trial registration

We will pre-register this study with the open science framework after article acceptance to ensure consistency between online registration and the published trial protocol.

Introduction

Depression is one the largest contributors to disability, estimated to affect about 280 million people worldwide [1]. There is a well-established evidence base that different forms of psychotherapy are effective in the treatment of depression [2–6]. Psychotherapy is also the preferred first line treatment option by many patients [7,8] and outperforms medication treatment in terms of side effects and long-term effectiveness [9–11]. However, the scalability of psychotherapy is limited due to several barriers, including high treatment costs, low availability of trained clinicians and stigma [12–14]. Therefore, the development of new treatment approaches and scalable formats is an urgent priority.

To meet this challenge, extensive research efforts have been conducted into digital behavioral interventions over recent decades [15,16]. Digital interventions are often self-help interventions that include psychotherapeutic content and exercises in the form of text, audio, video, and interactive elements in an app or on a website. Additional homework assignments and automated reminders or feedbacks are thought to promote behavior change and the integration of learned exercises into everyday life [17,18]. Digital interventions can be delivered as fully self-help interventions or include human guidance or support. The extent of human guidance can range from brief feedback on completed modules, clarifying of content and motivating participants (also referred to as guided self-help) to full parallel therapy sessions in face-to-face contact in form of blended care.

Although digital mental health is a relatively young field of research, there is already a well-established evidence base documenting the effectiveness of digital interventions in the prevention and treatment of depression [2,19]. The effect sizes of guided digital interventions are comparable to those of face-to-face therapies [2,20].

In addition to these positive results, there are other advantages of digital interventions including the ability to use interventions at a time that suits the user, scalability, and anonymity. In light of this, we see considerable efforts in many countries to implement digital interventions in the healthcare settings [21,22]. However, a much-debated question in research and practice is whether digital interventions are also suitable for people with depression who have suicidal thoughts and behavior (STB). STB are common in people suffering from depression and a key diagnostic criterium in both DSM-5 and ICD-11 [23–25].

The use of digital interventions in this population may raise some concerns, such as a lack of clinical impression of the patient, limited access to patients in crisis, and a potential delay in accessing established face-to-face treatments [26,27]. Additionally, a strong therapeutic relationship is thought to be a key component in the treatment of people with STB [28–30] which may be difficult to achieve to the same degree as in traditional face-to-face treatments when using digital formats.

Meta-analyses have demonstrated that digital interventions targeting suicidality ('direct interventions') can be effective in reducing suicidal ideation, with modest effect sizes [31–33]. Conversely, Torok and colleagues concluded in their meta-analysis of aggregated data that 'indirect interventions' (i.e., interventions that are directed towards targeting depression and only indirectly address suicidality) did not significantly reduce suicidal ideation [31]. However, this meta-analysis is limited in two respects. First, the studies included are not representative of digital interventions for depression, as only six out of more than eighty conducted trials were included [2]. The primary cause of exclusion was that most trials did not assess suicidal ideation with a specific diagnostic tool [31]. Second, in those six trials, elevated symptoms of

suicidality were not an inclusion criterion, which likely resulted in a sample where many participants did not experience suicidal ideation at baseline, thereby diminishing the potential to identify an effect on this outcome.

Meta-analyses of aggregated data are generally unable to investigate effects at the participant level or examine participant-level moderators, as they are confined to the reported group differences only [34,35].

Against this backdrop, we plan to conduct an individual patient data meta-analysis (IPDMA) to investigate treatment effects of digital interventions for depression STB.

IPDMAs merge the original research data from primary trials, allowing for more detailed and robust analyses of combined primary participant data on an item level [36,37].

Further, IPDMA provides several advantages over the analysis of aggregated data, such as the use of unreported data, integrity checks of trial IPD, standardization of outcome measures and participant characteristics, and risk of bias assessments based on trial IPD. An analysis of IPD also outperforms the analysis of aggregated data in terms of the range of outcome measures, the possibility of validity checks of analysis assumptions, the comparability by using a consistent method for the analysis, and the option to apply nonstandard effect measures [36].

The proposed IPDMA will build on an existing IPD pool on digital interventions for depression with a total of 62 included studies (14,088 participants) [20,38,39]. Currently, this dataset is undergoing a major update; thus, numbers are expected to alter. We will investigate treatment effects of digital depression interventions on STB, and to explore potential moderators of this effect.

Materials and Methods

This study is pre-registered with OSF (Note: We will pre-register this study with the open science framework after article acceptance to ensure consistency between online registration and the published trial protocol.) and is reported according to the PRISMA protocols statement [40]. The review will be reported according to the PRISMA IPD guideline [41]. We will report potential amendments to the protocol in the final report.

Eligibility criteria

Population: Studies will be eligible for inclusion if they focus on (a) adults or adolescents with (b) depressive symptoms either based on a cut-off score on self-report outcome measures or a diagnostic interview.

Interventions: Studies will be included if the experimental condition focuses on cognitive behavioral therapy for the reduction of depressive symptoms and is delivered via the web or a mobile application. Interventions may involve human support through the internet (either synchronous or asynchronous). We will exclude interventions that specifically target suicidal ideation; however, we will not exclude interventions that exclusively apply safety procedures or provide emergency contacts. Blended care, i.e., internet-based interventions combined with face-to-face treatment, will be excluded.

Comparators: Trials with the following control conditions will be eligible: usual care, attention control/psychological placebo, active control group, no intervention or waitlist.

Outcomes: Studies will be eligible if they report a quantitative measure of depressive symptoms. Depressive symptoms may be assessed by diagnostic interviews (e.g., Structured

Clinical Interview, SCID [42]), clinician rated scales (e.g., Hamilton Depression Rating Scale, HAMD [43]), or self-report scales (e.g., Beck Depression Inventory, BDI [44]) for depression. In addition, a quantitative measure of STB at baseline and post-intervention must be available in the published article and/or the IPD. STB may be measured via self-report, diagnostic interviews, or single items of depression scales (e.g., item 9 of the Patient Health Questionnaire 9, PHQ-9 [45]).

Studies: Primary studies must be randomized controlled trials (RCTs) published in a peer-reviewed journal. No restrictions on language or publication date will be applied.

Study identification and data extraction

We will use an already-existing database for RCTs investigating psychological interventions for depression that has been described in detail elsewhere [20,38,39]. The database is updated on an annual basis. IPD were sought regardless of whether the published articles reported the assessment of STB. We will extract deidentified IPD from all randomized participants, as well as relevant data items from published articles, including study identification items, study procedures, and aggregated clinical, and sociodemographic participant data. This will include data on the theoretical foundation of the intervention, delivery mode of content, amount of human support in remote treatments, and qualification of healthcare providers.

The primary outcome of this study will be the effects of the interventions on STB assessed by specific scales (e.g., Beck Scale for Suicide Ideation [46]), single item analysis from depression scales, or standardized interviews (e.g., suicidality item of the HAMD). Where available, we will use both specific scales and single items in separate analyses. If there are multiple validated measures of STB, we will prefer self-reports over clinician ratings, as individuals are

potentially more likely to report existing STB in self-report [47,48]. If there are several measures of the same hierarchy level within one study, we will either extract the measure most frequently used across eligible studies or select randomly if not evident.

Risk of bias

Risk of bias will be assessed with the RoB 2.0 (the revised Cochrane Risk of Bias Tool for randomized controlled trials) [49]. Bias arising from five domains will be assessed: from the randomization process, deviations from planned interventions, missing outcome data, measurement of the outcome, and from selection of the reported result. We will not evaluate the items referring to blinding of outcome assessors (signaling questions 4.3 to 4.5) since the tool defines participants as outcome assessors for outcomes that cannot be measured without incorporating the participant's perspective (also in clinician-rated outcomes), and blinding is rarely possible in psychotherapy research. By excluding these items from the evaluation, we avoid ceiling effects (i.e., overall high risk of bias for all studies) so that the ratings can distinguish between studies of high and low quality in this research field. The ratings will build on information from published trials and IPD. The information retrieved from IPD might alter the judgment of bias potential; for example, the choice of an appropriate and pre-registered analysis strategy in the IPDMA can lead to a lower risk of bias in selection of the reported result compared to the original trial. We will additionally check for potential range restrictions and high or low variances in the primary outcome. These aspects are especially relevant for single item measures.

Furthermore, we will examine availability bias. This refers to the question whether the studies included in this IPDMA (i.e., those that provide IPD, including a measure of STB) are representative for all relevant depression trials. First, we will perform a meta-analysis of

aggregated data with depressive symptoms as the outcome. We will perform a subgroup analysis for the studies included in this IPDMA vs. all other relevant trials that did not provide IPD including a measure of STB. The between-groups effect will be calculated using change scores from baseline. Second, we will display study characteristics for included trials vs. other relevant trials (e.g., year of publication, treatment format, number of sessions, age, gender, recruitment strategy, control condition) to identify any group differences.

Quality of evidence

Two independent reviewers will perform ratings of the quality of evidence concerning the treatment effect on STB using the Grading of Recommendations Assessment, Development and Evaluation (GRADE). We will evaluate the following domains: risk of bias, inconsistency, indirectness of evidence, imprecision, and publication bias. Any discrepancies will be resolved in discussion with a third researcher.

Statistical Analyses

STB will be the primary outcome of this study. However, STB is typically not the primary outcome of studies on depression interventions, which will present several methodological challenges. First, only a proportion of eligible studies will provide a validated measure of STB; most studies are likely to only provide single-item measures of STB as part of a depression measure. Single items with only few levels cannot be interpreted as continuous outcomes, limiting the range of potential analyses strategies. Second, individuals with STB and/or a history of suicide attempts are often excluded from depression trials. This floor effect limits the potential to show potential reductions in STB.

In the analyses, we will use all available data to provide an estimation of the effect on STB. We will conduct sensitivity analyses to check for the robustness of results. In addition, we will analyze STB in three indices: (1) a continuous score for the studies providing validated questionnaires for STB, (2) treatment response and (3) symptom deterioration for single-item measures. Severity of STB will be operationalized as continuous post-intervention scores. We will define treatment response vs. deterioration as an improvement vs. deterioration by at least one quartile from baseline on the relevant item. We chose not to use a 50% change criterion, because this would have equated a minor improvement in the low scale range with a larger improvement in the high scale range. Across different single-item measures, the potential to reach a one-point change might be different, which is why we will conduct sensitivity analyses for each measuring instrument (see sensitivity analyses below). All analyses will be conducted using R.

One-stage IPDMA

We will perform a one-stage IPDMA where all available IPD is merged in a hierarchical model, with participants nested in trials. This allows more sophisticated analyses compared to two-stage approaches where estimates are calculated for each study separately in a first step [50], and then pooled in a second step. Importantly, the one-stage approach allows fine-grained moderator analyses [51]. We will model a random intercept in each of the models, reflecting the hierarchical structure of the data. The treatment effect may be modeled as fixed or random (for a homogenous or heterogenous treatment effect, respectively); the model will be chosen based on model comparisons.

We will perform a multilevel linear regression to investigate the effects on severity of STB. This analysis will be conducted with the subgroup of studies providing a continuous measure

for STB. We will shift the scores to the same scale starting point and scale the post-intervention scores to the study-specific variance to ensure that scales are comparable across studies. In addition, we will control for baseline STB. We will exclude all participants that did not report STB at baseline from this analysis.

For the analyses of treatment response, we will conduct a multilevel logistic regression (response vs. no response). For this analysis, we will use single-item measures; this will also be the case for studies that additionally provide a full measure of STB to increase comparability across trials. We will exclude all participants that reported no baseline STB at baseline. Response will be defined as STB reduction of at least one point in the item at post-intervention.

For the analyses of symptom deterioration, we will conduct a multilevel logistic regression (deterioration vs. no deterioration), analogous to the analysis of treatment response. Thus, we will use single-item measures from all available studies. We will define deterioration as at least a one-point deterioration in the respective item from baseline to post-intervention per participant. Participants who report the maximum score in STB at baseline will be excluded from the analysis because they cannot show deterioration.

Furthermore, if feasible, we will investigate the effects on suicidal behavior using logistic multilevel regression. For this outcome, we will combine both suicide attempts and deaths to a binary outcome (i.e., either at least one suicide attempt during the intervention period, or no suicide attempt).

Two-stage IPDMA

In addition, we will perform two-stage IPDMAs to analyze the IPD separately in each study and then combine the estimates to calculate the pooled effect sizes. We will conduct a separate two-stage IPDMA for all three indices of STB. Thus, we will calculate Hedges' g for the severity of STB, as well as the log odds ratios of treatment response and symptom deterioration. Based on the analysis of treatment response, we will calculate the number-needed-to-treat (NNT). This procedure will allow for comparisons of the proposed IPDMA with prior and future meta-analyses. We will investigate between-study heterogeneity using I2.

Moderators of STB severity

If feasible, we will investigate whether characteristics of studies, interventions, or participants modify the effects on STB. These analyses will be conducted in the one-stage IPDMA. We will perform moderator analyses with the following variables if feasible across the identified trials: (a) clinical variables at baseline and changes-from-baseline, including depressive symptoms, hopelessness, anxiety, alcohol use and psychiatric medication, (b) sociodemographic variables, including age, gender, level of education, relationship status, employment status, treatment history, and current or previous side treatments, (c) study-level variables including human support in the interventions, treatment duration, number of modules, and type of control condition.

Sensitivity analyses

We will conduct the following sensitivity analyses: First, we will exclude all trials at overall high risk of bias. Second, we will exclude trials with sample sizes below the median to investigate potential small-study effects. Third, we will conduct the single-item analyses (i.e., response and deterioration) for each single-item measure separately (when used several times across

studies) to identify whether the findings are robust across different measures. Thus, we will be able to detect potential differences across measures which might, for example, stem from a different number of levels in the items. Fourth, the single-item analyses on symptom improvement and deterioration will be conducted with all participants.

Discussion

Depression remains a major challenge to healthcare systems worldwide and digital interventions are considered as one scalable treatment option especially when first-line treatments are not desired or available. However, digital interventions for depression are not commonly recommended for people with self-harm behavior.

The results of this IPDMA will synthesize relevant data to provide information about the impact of digital interventions for depression on STB. A key strength of the IPDMA method in this context is its capacity to capture data on STB from single items of depression scales that may not be reported in the original publications. The planned analyses on moderators of this effect will provide insight into potential beneficial or harmful effects in particular subgroups of patients, thereby contributing to the informed selection of digital interventions for depression.

This investigation has some limitations that should be acknowledged. Firstly, specific questionnaires on STB are expected to be only available in a few trials, meaning that the main analyses will focus on single items extracted from depression scales (e.g., item 9 of the PHQ-9). This reduces the degree of granulation, as well as increasing the error of the effect size estimation, and consequently reduces the content validity. Secondly, individuals who report

elevated STB at baseline are often excluded from randomized clinical trials [27], which reduces the baseline symptom severity in the study population and therefore leaves less room for improvement compared to trials where STB are the primary outcome. Additionally, we will exclude people without STB at baseline from our response analysis, further reducing the total number of individuals in the planned analysis.

Despite these limitations, this IPDMA is the first meta-analytic investigation to analyze the effects of digital depression interventions on STB in most available trials. The results of this study will provide invaluable insights into the benefits and risks of delivering digital interventions for people who experience suicidal thoughts in the context of depression.

Acknowledgements

The study described in this protocol is funded by the German Research Foundation (project "*Effects of internet interventions for depression on suicide ideation - a meta-analysis of individual participant data (IPDMA), grant No. SA 3767/2-1).* The funders had no role in the study design, the decision to publish, or in the preparation of the manuscript. The views expressed are those of the author(s) and not necessarily those of the funder. The article processing charge was funded by the German Research Foundation (DFG) and the University of Freiburg in the funding program Open Access Publishing.

Competing interests

The authors have declared that no competing interests exist.

Authors' Contributions

LBS is the overall guarantor of this research protocol and takes responsibility for the content. LBS designed the trial and acquired funding together with HB. LBS acquired ethics approval, is the chief investigator of the trial, coordinates the trial management and wrote and revised the manuscript. RB, MB and PD made major contributions to methodological aspects. EK and PC will provide access to the IPD database. EK, WVB, PC, TT, MD and HB added their expertise to the study design and methodology. All authors provided valuable revisions and approved the final version of the manuscript.

References

1. World Health Organization. World mental health report: Transforming mental health for all. Geneva; 2022.
2. Moshe I, Terhorst Y, Philippi P, Domhard M, Cuijpers P, Cristea I, et al. Digital interventions for the treatment of depression: A meta-analytic review. Psychol Bull. 2021;147: 749–786. doi:10.1037/bul0000334
3. Barth J, Munder T, Gerger H, Nüesch E, Trelle S, Znoj H, et al. Comparative Efficacy of Seven Psychotherapeutic Interventions for Patients with Depression: A Network Meta-Analysis. PLoS Med. 2013;10. doi:10.1371/journal.pmed.1001454
4. Cuijpers P, Huibers M, Ebert DD, Koole SL, Andersson G. How much psychotherapy is needed to treat depression? A metaregression analysis. J Affect Disord. 2013;149: 1–13. doi:10.1016/j.jad.2013.02.030
5. Cuijpers P, Karyotaki E, Eckshtain Di, Ng MY, Corteselli KA, Noma H, et al. Psychotherapy for Depression across Different Age Groups: A Systematic Review and Meta-analysis. JAMA Psychiatry. 2020; 1–9. doi:10.1001/jamapsychiatry.2020.0164
6. Zhou X, Hetrick SE, Cuijpers P, Qin B, Barth J, Whittington CJ, et al. Comparative efficacy and acceptability of psychotherapies for depression in children and adolescents: A systematic review and network meta-analysis. World Psychiatry. 2015;14: 207. doi:10.1002/WPS.20217
7. McManus S, Bebbington P, Jenkins R, Brugha T. Mental health and wellbeing in England: Adult Psychiatric Morbidity Survey 2014. Leed, UK; 2016.
8. McHugh RK, Whitton SW, Peckham AD, Welge JA, Otto MW. Patient preference for psychological vs pharmacologic treatment of psychiatric disorders: A meta-analytic review. Journal of Clinical Psychiatry. 2013. pp. 595–602. doi:10.4088/JCP.12r07757
9. Almohammed OA, Alsalem AA, Almangour AA, Alotaibi LH, Al Yami MS, Lai L. Antidepressants and health-related quality of life (HRQoL) for patients with depression: Analysis of the medical

expenditure panel survey from the United States. PLoS One. 2022;17: e0265928. doi:10.1371/JOURNAL.PONE.0265928

10. Dobson KS, Hollon SD, Dimidjian S, Schmaling KB, Kohlenberg RJ, Gallop RJ, et al. Randomized trial of behavioral activation, cognitive therapy, and antidepressant medication in the prevention of relapse and recurrence in major depression. J Consult Clin Psychol. 2008;76: 468–477. doi:10.1037/0022-006X.76.3.468

11. Karyotaki E, Smit Y, Holdt Henningsen K, Huibers MJH, Robays J, De Beurs D, et al. Combining pharmacotherapy and psychotherapy or monotherapy for major depression? A meta-analysis on the long-term effects. Journal of Affective Disorders. Elsevier; 2016. pp. 144–152. doi:10.1016/j.jad.2016.01.036

12. Patel V, Chowdhary N, Rahman A, Verdeli H. Improving access to psychological treatments: Lessons from developing countries. Behaviour Research and Therapy. 2011;49: 523–528. doi:10.1016/J.BRAT.2011.06.012

13. Saraceno B, van Ommeren M, Batniji R, Cohen A, Gureje O, Mahoney J, et al. Barriers to improvement of mental health services in low-income and middle-income countries. Lancet. 2007. pp. 1164–1174. doi:10.1016/S0140-6736(07)61263-X

14. World Health Organization. Mental health atlas 2020. Geneva; 2021. Available: https://www.who.int/publications/i/item/9789240036703

15. Torous J, Myrick KJ, Rauseo-Ricupero N, Firth J. Digital mental health and COVID-19: Using technology today to accelerate the curve on access and quality tomorrow. Journal of Medical Internet Research. 2020. doi:10.2196/18848

16. Moshe I, Terhorst Y, Opoku Asare K, Sander L, Ferreira D, Baumeister H, et al. Predicting Symptoms of Depression and Anxiety Using Smartphone and Wearable Data. Front Psychiatry. 2021;12. doi:10.3389/fpsyt.2021.625247

17. Andersson G. Using the Internet to provide cognitive behaviour therapy. Behaviour Research and Therapy. 2009;47: 175–180. doi:10.1016/j.brat.2009.01.010

18. Singla DR, Raviola G, Patel V. Scaling up psychological treatments for common mental disorders: a call to action. World Psychiatry. 2018;17: 226–227. doi:10.1002/wps.20532

19. Rigabert A, Motrico E, Moreno-Peral P, Resurrección DM, Conejo-Cerón S, Cuijpers P, et al. Effectiveness of online psychological and psychoeducational interventions to prevent depression: Systematic review and meta-analysis of randomized controlled trials. Clin Psychol Rev. 2020;82. doi:10.1016/j.cpr.2020.101931

20. Karyotaki E, Ebert DD, Donkin L, Riper H, Twisk J, Burger S, et al. Do guided internet-based interventions result in clinically relevant changes for patients with depression? An individual participant data meta-analysis. Clin Psychol Rev. 2018;63: 80–92. doi:10.1016/j.cpr.2018.06.007

21. Titov N, Dear B, Nielssen O, Staples L, Hadjistavropoulos H, Nugent M, et al. ICBT in routine care: a descriptive analysis of successful clinics in five countries. Internet Interv. 2018;13: 108–115. doi:10.1016/j.invent.2018.07.006

22. Lattie EG, Stiles-Shields C, Graham AK. An overview of and recommendations for more accessible digital mental health services. Nature Reviews Psychology. 2022;1: 87–100. doi:10.1038/s44159-021-00003-1

23. American Psychiatric Association. Diagnostic and statistical manual of mental disorders. 5th ed. London: American Psychiatric Pub; 2013.

24. World Health Organization. ICD-11: International classification of diseases (11th revision). 2019. Available: https://icd.who.int/

25. Rihmer Z. Suicide risk in mood disorders. Curr Opin Psychiatry. 2007;20: 17–22. doi:10.1097/YCO.0b013e3280106868

26. Mishara BL, Weisstub DN. Ethical and legal issues in suicide research. Int J Law Psychiatry. 2005;28: 23–41. doi:10.1016/j.ijlp.2004.12.006

27. Sander L, Gerhardinger K, Bailey E, Robinson J, Lin J, Cuijpers P, et al. Suicide risk management in research on internet-based interventions for depression: a synthesis of the current state and recommendations for future research. J Affect Disord. 2020;263: 676–683. doi:10.1016/j.jad.2019.11.045

28. Joiner TE, Van Orden KA, Witte TK, Rudd MD. The interpersonal theory of suicide: Guidance for working with suicidal clients. Washington, DC: American Psychological Association; 2009. doi:doi.org/10.1037/11869-000

29. Rudd MD, Brown GK. A cognitive theory of suicide: Building hope in treatment and strengthening the therapeutic relationship. Building a therapeutic alliance with the suicidal patient. American Psychological Association; 2011. pp. 169–181. doi:10.1037/12303-010

30. Dunster-Page C, Haddock G, Wainwright L, Berry K. The relationship between therapeutic alliance and patient's suicidal thoughts, self-harming behaviours and suicide attempts: A systematic review. J Affect Disord. 2017;223: 165–174. doi:10.1016/j.jad.2017.07.040

31. Torok M, Han J, Baker S, Werner-Seidler A, Wong I, Larsen ME, et al. Suicide prevention using self-guided digital interventions: A systematic review and meta-analysis of randomised controlled trials. Lancet Digit Health. 2020;2: E25–E36. doi:10.1016/S2589-7500(19)30199-2

32. Büscher R, Torok M, Terhorst Y, Sander LB. Internet-based cognitive behavioral therapy (iCBT) to reduce suicidal ideation: A systematic review and meta-analysis. JAMA Netw Open. 2020;3: e203933. doi:10.1001/jamanetworkopen.2020.3933

33. Büscher R, Beisemann M, Doebler P, Micklitz HM, Kerkhof A, Cuijpers P, et al. Digital cognitive–behavioural therapy to reduce suicidal ideation and behaviours: A systematic review and meta-analysis of individual participant data. Evidence Based Mental Health. 2022;25: e8–e17. doi:10.1136/ebmental-2022-300540

34. Weitz E, Hollon SD, Kerkhof A, Cuijpers P. Do depression treatments reduce suicidal ideation? the effects of CBT, IPT, pharmacotherapy, and placebo on suicidality. J Affect Disord. 2014;167: 98–103. doi:10.1016/j.jad.2014.05.036

35. Cuijpers P, De Beurs DP, van Spijker BAJ, Berking M, Andersson G, Kerkhof AJFM. The effects of psychotherapy for adult depression on suicidality and hopelessness: a systematic review and meta-analysis. J Affect Disord. 2013;144: 183–190. doi:10.1016/j.jad.2012.06.025

36. Tierney JF, Vale C, Riley R, Smith CT, Stewart L, Clarke M, et al. Individual participant data (IPD) meta-analyses of randomised controlled trials: Guidance on their use. PLoS Med. 2015;12: e1001855. doi:10.1371/journal.pmed.1001855

37. Stewart L, Tierney J, Clarke M. Chapter 19: reviews of individual patient data. In: Higgins JP, Green S, editors. Cochrane Handbook for Systematic Reviews of Interventions Version 510. The Cochrane Collaboration; 2011.

38. Karyotaki E, Kemmeren L, Riper H, Twisk J, Hoogendoorn A, Kleiboer A, et al. Is self-guided internet-based cognitive behavioural therapy (iCBT) harmful? An individual participant data meta-analysis. Psychol Med. 2018;48: 2456–2466. doi:10.1017/S0033291718000648

39. Karyotaki E, Riper H, Twisk J, Hoogendoorn A, Kleiboer A, Mira A, et al. Efficacy of self-guided internet-based cognitive behavioral therapy in the treatment of depressive symptoms: a meta-analysis of individual participant data. JAMA Psychiatry. 2017;74: 351–359. doi:10.1001/JAMAPSYCHIATRY.2017.0044

40. Shamseer L, Moher D, Clarke M, Ghersi D, Liberati A, Petticrew M, et al. Preferred reporting items for systematic review and meta-analysis protocols (PRISMA-P) 2015: elaboration and explanation. BMJ. 2015;350: g7647. doi:doi.org/10.1136/bmj.g7647

41. Stewart LA, Clarke M, Rovers M, Riley RD, Simmonds M, Stewart G, et al. Preferred reporting items for a systematic review and meta-analysis of individual participant data: The PRISMA-IPD statement. JAMA - Journal of the American Medical Association. 2015;313: 1657–1665. doi:10.1001/jama.2015.3656

42. First M, Williams J, Karg R, Spitzer R. Structured Clinical Interview for DSM-5 - Research Version (SCID-5-RV). Arlington, VA: American Psychiatric Association; 2015. doi:10.1002/9781118625392.wbecp351

43. Hamilton M. A rating scale for depression. J Neurol Neurosurg Psychiatry. 1960;23: 56–63. doi:10.1136/jnnp.23.1.56

44. Beck AT, Ward CH, Mendelson M, Mock J, Erbaugh J. An inventory for measuring depression. Arch Gen Psychiatry. 1961;4: 561–571. doi:http://dx.doi.org/10.1001/archpsyc.1961.01710120031004

45. Löwe B, Kroenke K, Herzog W, Gräfe K. Measuring depression outcome with a brief self-report instrument: Sensitivity to change of the Patient Health Questionnaire (PHQ-9). J Affect Disord. 2004;81: 61–66. doi:10.1016/S0165-0327(03)00198-8

46. Beck AT, Steer RA. Manual for the Beck Scale for Suicide Ideation. San Antonio: Psychological Corporation; 1991.

47. Velting DM, Rathus JH, Asnis GM. Asking Adolescents to Explain Discrepancies in Self-Reported Suicidality. Suicide Life Threat Behav. 1998;28: 187–196. doi:10.1111/J.1943-278X.1998.TB00638.X

48. Kaplan ML, Asnis GM, Sanderson WC. Suicide assessment: Clinical interview vs. self-report. J Clin Psychol. 1994;50: 294–298. doi:https://doi.org/10.1002/1097-4679(199403)50:2

49. Higgins J, Sterne JAC, Savovic J, Page M, Hróbjartsson A, Bourtron I, et al. A revised tool for assessing risk of bias in randomized trials. Cochrane Database of Systematic Reviews. 2016;10: 29–31. doi:10.1002/14651858.CD201601

50. Burke DL, Ensor J, Riley RD. Meta-analysis using individual participant data: one-stage and two-stage approaches, and why they may differ. Stat Med. 2017;36: 855–875. doi:10.1002/sim.7141

51. Berlin JA, Santanna J, Schmid CH, Szczech LA, Feldman HI. Individual patient- versus group-level data meta-regressions for the investigation of treatment effect modifiers: ecological bias rears its ugly head. Stat Med. 2002;21: 371–387. doi:10.1002/sim.1023

PRISMA-P Checklist

Click here to access/download
Supporting Information
PRISMA_P_CHECKLIST.pdf
